# Biologically-Inspired Pulse Signal Processing for Intelligence at the Edge

**DOI:** 10.3389/frai.2021.568384

**Published:** 2021-09-08

**Authors:** Kan Li, José C. Príncipe

**Affiliations:** ^1^ Computational NeuroEngineering Laboratory (CNEL), Department of Electrical and Computer Engineering, University of Florida, Gainesville, FL, United States

**Keywords:** automatic speech recognition, edge computing, internet of things, keyword spotting, kernel adaptive filtering, kernel method, reproducing kernel hilbert space, neuromorphic computation

## Abstract

There is an ever-growing mismatch between the proliferation of data-intensive, power-hungry deep learning solutions in the machine learning (ML) community and the need for agile, portable solutions in resource-constrained devices, particularly for intelligence at the edge. In this paper, we present a fundamentally novel approach that leverages data-driven intelligence with biologically-inspired efficiency. The proposed Sparse Embodiment Neural-Statistical Architecture (SENSA) decomposes the learning task into two distinct phases: a training phase and a hardware embedment phase where prototypes are extracted from the trained network and used to construct fast, sparse embodiment for hardware deployment at the edge. Specifically, we propose the Sparse Pulse Automata via Reproducing Kernel (SPARK) method, which first constructs a learning machine in the form of a dynamical system using energy-efficient spike or pulse trains, commonly used in neuroscience and neuromorphic engineering, then extracts a rule-based solution in the form of automata or lookup tables for rapid deployment in edge computing platforms. We propose to use the theoretically-grounded unifying framework of the Reproducing Kernel Hilbert Space (RKHS) to provide interpretable, nonlinear, and nonparametric solutions, compared to the typical neural network approach. In kernel methods, the explicit representation of the data is of secondary nature, allowing the same algorithm to be used for different data types without altering the learning rules. To showcase SPARK’s capabilities, we carried out the first proof-of-concept demonstration on the task of isolated-word automatic speech recognition (ASR) or keyword spotting, benchmarked on the TI-46 digit corpus. Together, these energy-efficient and resource-conscious techniques will bring advanced machine learning solutions closer to the edge.

## 1 Introduction

Machine Learning (ML), especially deep learning (DL), is rapidly becoming the *de facto* model-based solution in many areas of information technologies because of its unprecedented accuracy in many practical problems, such as image classification, speech recognition, and natural language processing, etc (Collobert and Weston, 2008; Hinton et al., 2012; Krizhevsky et al., 2012). However, this breakthrough in performance does not come for free, i.e., the no-free-lunch theorem (Wolpert, 1996). DL solutions are typically fueled by enormous amounts of data (where data is used as a cheap prior for modeling) and need high computational load and memory usage due to their large multiscale architectures, which require specialized hardware, e.g., graphics processing units (GPUs) and/or cloud computing.

In many engineering applications, from monitoring to process control, as well as point of sales, inventory management, speech recognition, real-time decision making and monitoring in medical applications, automatic translation, social media, etc., one needs to deliver solutions that can be run in small microprocessors or embedded systems. Perhaps the application domain that suffers the most regarding the high computational demand of DL is edge computing (Shi et al., 2016), i.e., the ability to implement intelligent, online signal processing solutions near the sensors, saving tremendous bandwidth and reducing latency by transmitting processed decisions instead of raw data. Likewise, the implementation of Industry 4.0 standards that are revolutionizing the manufacturing and industrial platforms will benefit from the same approach. These, along with ethical, privacy, and security issues will only get worse if not addressed properly, as more and more devices become connected. An estimated 29.3 billion networked devices will be connected to IP networks in 2023 (Cisco, 2020), and roughly 850 Zettabytes (ZB) of data will be generated annual outside the cloud by 2021, competing for the global data center traffic of only 21 ZB (Cisco, 2019).

To deliver real-time machine learning solutions, we should not forget Widrow’s least mean square (LMS) algorithm, invented in 1960 (Widrow and Stearns, 1985), which modifies system parameters after every data sample. If one can accomplish this update before the next data sample comes in (determined by the input sampling frequency), we have real-time learning on the linear model. With the advent of kernel adaptive filtering (KAF) (Liu et al., 2010), real-time learning on the nonlinear model has been achieved. We more recently advanced the state-of-the-art by proposing the first fully-developed state-space model implementation in the Reproducing Kernel Hilbert Space (RKHS), an adaptive dynamical system called the Kernel Adaptive AutoRegressive-Moving-Average (KAARMA) algorithm, using the representer theorem (Li and Príncipe, 2016). Recurrent mappers are essential for time series applications. We know how to build large-scale solutions from the ground up, but the question remains on how to effectively scale them down for the edge, where complex ML algorithms and advanced electronic hardware are not supported.

The proposed approach will be novel in two fundamental respects. First, we posit that for many applications, especially in offline learning or implementation, there is no good reason, besides straightforwardness, to deploy the same ML program that searches in a training set for the optimum of the decision boundaries (or fitting hyperplanes), which has been embraced by the ML community. Once the parameters are fixed, the input-output mapping has been discovered (i.e., deterministic) and can be easily approximated with a sparse embodiment in hardware, e.g., a field programmable gate array (FPGA), using memory-based techniques augmented with rule-based finite state machines (FSMs) or automata, akin to lookup tables. Second, following an inspiration from biology, there must exist advantages of power and bandwidth in working with point processes, because the neurons in the brain interact in this way. Spike-based computation has been actively researched in neuromorphic computing (Maass et al., 2005), however we submit that the critical issue is how to effectively train these architectures, even if we overlook the many other bottlenecks of digital computation (still heavy) or analog implementations (still plagued by parameter drift). Currently, only the projections are trained, which is a shortcoming. Alternatively, our approach calls for processing with spike or pulse trains in the time domain, the essence of neuromorphic solutions, but using statistical signal processing and machine learning to find the optimal solutions, which solves both shortcomings of neuromorphic approaches. We call this approach the Sparse Embodiment Neural-Statistical Architecture (SENSA), which will be automated with the learned (fixed) input-output mapping for edge computing and Internet of Things (IoT).

We prefer to employ the terminology “pulse trains” instead of the neuromorphic “spike trains” because of the specific way we convert continuous-time-and-amplitude signals created in the real world with an integrate-and-fire converter (IFC) (Singh Alvarado et al., 2011). We showed mathematically that the IFC creates a discrete (positive and negative pulses) continuous-time pulse train where the time distance between pulses (inter-pulse interval) translates the area under the curve and allows the reconstruction of the original continuous signal with an arbitrarily small approximation error (Feichtinger et al., 2012). Subsequently, we have shown that it is possible to perform arithmetic operations (addition, multiplication, and convolution) of analog signals directly using pulse trains (Nallathambi and Principe, 2020). The big appeal of this approach is the potential ultra-low power implementation, because of the asynchronous nature of the computation, i.e., algorithm computes only when there is a pulse. Hence, theoretically, this only requires flip-flops and memory, not synchronous processors. One of the difficulties faced in our early work was the need to design automata using expert knowledge about the signal. This difficulty was subsequently removed with the use of a ML methodology that learns directly from pulse trains the features needed for the application using supervised learning. Our approach is online and utilizes segments of the multichannel pulse trains to define a reproducing kernel, then maps the pulse data to an RKHS, where classical linear methods are used to derive nonlinear solutions in the original input space using inner products.

Specifically, we propose the Sparse Pulse Automata via Reproducing Kernel (SPARK) method for SENSA, which first constructs a learning machine in the form of a dynamical system using energy efficient pulse trains and KAARMA, then extracts a sparse rule-based solution, in the form of automata using metric-based prototypes, for rapid deployment in edge computing platforms. The theory of reproducing kernels (Aronszajn, 1950) is an attractive alternative to the ubiquitous neural networks used in DL, as it is a more principled and systematic approach to training learning machines. There is no architectural hyperparameter to set (i.e., nonparametric), and, in the case of feedforward solutions such as support vector machines (SVMs), global optimum can be achieved directly from the data using quadratic programming. The results can also be readily interpreted using geometry and statistical learning theory. The recent resurgence of interest in kernel methods has paved the way for competitive performances on many challenging tasks compared to deep neural networks (Rahimi and Recht, 2007; Cho and Saul, 2009; Huang et al., 2014; Wilson et al., 2016).

Edge computing can benefit from a kernel perspective, making them more powerful and applicable to nonlinear processing in a simpler and more elegant way. To overcome the shortcoming of conventional kernel methods which required fixed (constant) dimension inputs or static patterns, we developed the KAARMA algorithm for sequence learning and to account for the variability in the temporal dimension of spatiotemporal signals (Li and Príncipe, 2016).

The key advantage of kernel methods is their ability to work with functions in the RKHS, and changing the reproducing kernel function has no impact on the underlying learning algorithm. Therefore, SPARK is agnostic to the input type and can operate on real vectors using the Gaussian kernel, or directly on event-based pulse trains, by designing an appropriate pulse kernel. This interchangeability is not applicable to artificial neural networks (ANNs) and spiking neural networks (SNNs) because spike trains are non-differentiable.

As a proof-of-concept, we will apply the SENSA paradigm to isolated speech recognition using SPARK. Speech signals are highly non-stationary with large variations in their spatiotemporal realizations. We have already shown that KAARMA outperforms hidden Markov models (HMMs) and liquid state machines (LSMs) for spoken digit recognition (Li and Príncipe, 2018a) using conventional speech features and pulse trains mimicking the cochlea of the human auditory system, in terms of improved accuracy and noise robustness. Our pulse-based neural-statistical approach is ideal for IoT and edge computing, as the only requirement is time stamping at the Nyquist frequency of the data source, which decreases the hardware implementation clock rate, such as in an FPGA, by orders of magnitude compared with the current generation of digital signal processors (DSPs) and GPUs. We will demonstrate that this combination of biologically-inspired pulse input and memory-based implementation further reduces the computational cost and footprint, which opens the door for realtime microwatt intelligent signal processing in practical intelligence at the edge applications.

We organize the remainder of this paper as follows. In Section 2, we present the Sparse Embodiment Neural-Statistical Architecture and SPARK. Performances on isolated word recognition in both software and hardware are evaluated in Section 3 using the benchmark TI-46 digit corpus. Finally, Section 4 concludes this paper.

## 2 Methods

SENSA consists of two distinct phases. First, we encode the time structure and temporal evolution of time series, such as speech, in spike or pulse trains. The energy efficiency of neural signal transmission plays an integral role in biology, particularly in the information processing of the human brain. Specifically, we wish to develop a biologically-inspired end-to-end ML system, without hand-designed feature extraction or engineering, past the stage of pulse generation.

Furthermore, rather than learning static feedforward models, we aim to learn and model the dynamics directly on the pulse trains by constructing a state-space model (SMM) in the RKHS. This dynamical system approach is rooted in classical Newtonian mechanics, where the evolution of the observables over time is governed by attractors, grammar or rules. This framework can be used to explain seemingly-chaotic time series using simple latent state transitions. Grammatical inference or rule discovery of dynamical systems provides a parsimonious way to analyze, model, and classify trajectories with large variations in realization (time and scale) but are equivalent in dynamics. This enables us to find such elegant attracting behavior and scale down ML solutions.

Recurrent networks are typically used for learning time structures and temporal dependencies in data. However, in practice, the solution depends on the specific data type, e.g., conventional ANN and SNN involve completely different learning mechanisms and output, due to the non-differentiable activation functions of spiking neurons. In this paper, we strive for a unifying framework that is independent of input signal representation. This vastly improves the usefulness and versatility of the ML solutions. To achieve this goal, we apply the theory of RKHS to map the pulse-train input (single or multichannel) into an RKHS, then construct and learn a dynamical system in this space using KAARMA. For kernel methods, the learning algorithm is defined in terms of the inner products between potentially infinite-dimensional features or functions, in the functional space, and not in terms of the original input representation. The input can take the form of discrete pulses, continuous-valued attributes, symbols, or graphs. We can compute the inner product in closed form using the *kernel trick*. Therefore, we have the freedom to select the input representation independently with an appropriate reproducing kernel function, and the learning algorithm is not impacted when changing the input-kernel pair.

Second, we formulate a systematic embedment method that extracts the learned dynamics in the form of simple FSMs or lookup tables using prototypes and implement or deploy them in hardware. Once the dynamics are learned and fixed, these memory-based solutions require no complex computation and completely replace the state transition and observation functions of the dynamical system in the previous phase. These sparse embodiments are ideal for rapid deployment in IoT and edge computing, where resources are limited. This specific formulation of SENSA using kernel methods is termed SPARK.

### 2.1 Sparse Embodiment Neural-Statistical Architecture

Conventional ML uses the same network for training and testing. However, there are many scenarios where complex, large-scale learning cannot be supported, such as in edge computing. SENSA is an approach that maintains a large network, trained either online or offline, always using pulse-encrypted data, and periodically deploys or updates an agile portable solution, a scaled-down version approximated to the desired accuracy, for realtime processing near the sensors in resource-constrained environments, using memory-based techniques augmented with rule-based deterministic finite automata (DFA).

The energy efficiency of computing with spike trains has been long understood and appreciated (Maass, 1997; Furber et al., 2014; Merolla et al., 2014), particularly in the context of spiking neural networks. However, there exists a chasm between the methodologies for continuous amplitude signals and event-based spike trains. SNNs trained either directly using spiking-timing dependent plasticity (STDP) or through back-propagation have not demonstrated comparable results to DL ANNs. Current research in neuromorphic architectures mostly focuses instead on converting a trained deep neural network to an SNN to improve its accuracy (Rueckauer et al., 2017). This gap can be easily bridged with RKHS methodologies, since the actual input representation is secondary for kernel methods. Indeed, the same ML code can be utilized for both types of signals, once the proper kernel function for each signal modality is chosen. Thus, we propose an alternative neural-statistical approach that uses spike or pulse input to derive energy efficient and low latency solutions. This novel approach enables ultra-low power deployment in portal devices connected to intelligent personal assistants and other IoT devices.

**FIGURE 1:**
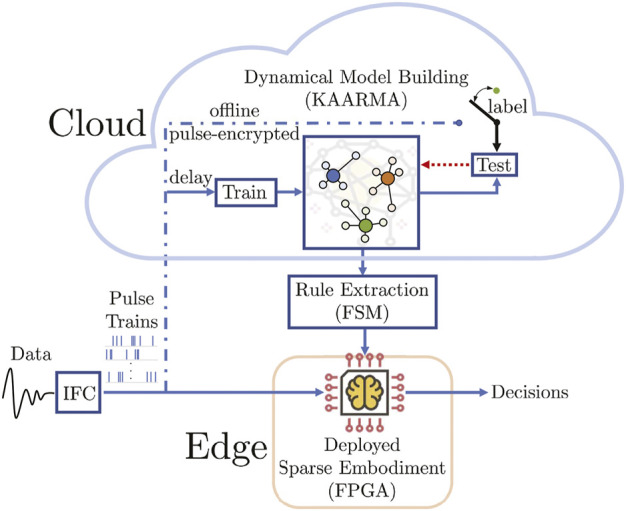
Sparse Embodiment Neural-Statistical Architecture (SENSA): once learning parameters are trained, the input-output mapping can be easily approximated using memory-based techniques augmented with rule-based finite state machine (FSM) for rapid deployment in hardware (FPGA). We call this specific formulation using kernel methods for model building Sparse Pulse Automata via Reproducing Kernel (SPARK).

As illustrated in Figure 1, this novel, hybrid neuromorphic-statistical ML approach for brain-inspired learning first converts analog signals into data-efficient binary pulse trains, then uses kernel method to train adaptive state-space models. These dynamical models can then be approximated using sparse embodiment and deployed in a network of reprogrammable finite state machines, using only the timing of the digital pulses and table lookups to index the next state or output, without performing expensive computations. Compared with conventional input features, event-based pulse trains (used extensively in the nervous system as the primary mode of information processing and transmission) are highly efficient and robust to noise, leading to extremely small hardware footprint which are ideally suited for low power on-chip processing. Furthermore, by leveraging FSMs, the proposed approach requires no arithmetic for functional evaluation (output values are retrieved from memory), allowing complex processing capability in real-time that would otherwise entail prohibitive expenses or be pushed away from the edge and into the cloud. Thus, SENSA offers increased security and privacy by eliminating the need to continuously upload potentially sensitive data online for processing, and uses only the pulse-encrypted data.

We choose to use kernel methods instead of the typical neural networks used in DL, because the theory of RKHS is a theoretically-grounded, powerful, and versatile unifying framework for solving nonlinear problems in signal processing and ML. There is no *a priori* need to choose the proper number of parameters for modeling. The complexity of kernel methods scales with the number of training instances, not the dimensionality of the input features or the architecture of the network. Thus, kernel methods are extremely effective when the data dimension is high and the number of training samples is relatively small. Furthermore, all online kernel methods lend naturally to sparsification techniques as data samples are processed on an individual basis. We have developed several techniques to curb the growth of similar networks, including using a combination of novelty and surprise-based criteria and quasi-orthogonal decomposition (Li and Príncipe, 2016; Li and Príncipe, 2017; Li and Príncipe, 2018a). The biggest advantage of SPARK is that the actual data representation is secondary to the learning algorithm. This key feature imposes no restriction on the relationships between the input signals or their types, e.g., we can easily model a biological systems using a combination of continuous-amplitude local field potentials (LFPs), spike-train neural signal, and vectorized state variables at the input. This makes SPARK solutions extremely flexible.

### 2.2 Model Building Using Kernel Adaptive AutoRegressive-Moving-Average

We submit that dynamical approaches are much more appropriate to deal with spatiotemporal signals, and when coupled with pulse trains, either synthesized or generated directly from activity-driven, event-based vision sensors, a compressive sampling approach is achieved that decreases the correlation over time which plagues random processes analysis (and it is still unappreciated). Capturing structure in time series requires nonlinear state models. State modeling represents the signal history very efficiently and exploits this information at the current time. Our approach uses nonlinear data projections to an infinite-dimensional Hilbert space of functions, which we termed kernel adaptive filtering (Liu et al., 2010). The theory of RKHS simplifies the operations on functions by restricting them to inner products (the *kernel trick*).

Here, we briefly introduce the basic concept of linear filtering in the RKHS or KAF. For a set of *N* data points $D={\left\{x_{i},y_{i}\right\}}_{i=1}^{N}$, with input $x_{i}\in R^{n_{x}}$ (where $R^{n_{x}}$ is the *n*_*x*_-dimensional real space) and output $y_{i}\in R$, we want to infer the underlying function *y* = *f*(**x**). From a weight-space perspective, the estimated latent function $\hat{f}\left(x\right)$ is expressed in terms of a set of parameters or weight vector $W\in R^{n_{x}}$ as

$$\hat{f}\left(x\right)=W^{⊺}x.$$ (1)

To overcome the limited expressiveness of this linear model, we first project the input vector $x\in U\subseteq R^{n_{x}}$ (where U is a compact input domain in $R^{n_{x}}$) into a potentially infinite-dimensional feature space F, using a U→F mapping *ϕ*(⋅), then Eq. 1 becomes

$$\hat{f}\left(x\right)={\left\langle \Omega ,\phi \left(x\right)\right\rangle }_{F}=\Omega^{⊺}\phi \left(x\right),$$ (2)

where **Ω** is a potentially infinite dimensional weight vector in the feature space, denoted by Greek letter.

Applying the *kernel trick* and the representer theorem (Scholkopf et al., 2001), Eq. 2 can be expressed as a weighted sum of basis functions

$$\hat{f}\left(x\right)=\overset{N}{\underset{i=1}{\sum }}\alpha_{i}K\left(x_{i},x\right),$$ (3)

where *α*_*i*_ are the coefficients, $K\left(x,x^{\prime }\right)$ is a reproducing or Mercer kernel associated with the inner product ${\left\langle \phi \left(x\right),\phi \left(x^{\prime }\right)\right\rangle }_{F}$, and *N* is the number of basis functions or training samples. Note that F is equivalent to the RKHS H induced by the kernel if we identify ϕ(x)=K(x,⋅), i.e., F=H. Typically, the Gaussian radial basis function (RBF) is used

$$K_{a}\left(x,x^{\prime }\right)=\exp\left(-a\|x-x^{\prime }{\|}^{2}\right),$$ (4)

with the kernel parameter *a* > 0. The key feature of Mercer kernels is the universal approximation property: an arbitrary continuous target function can be approximated uniformly to any degree of accuracy over any compact subset of the input space. The theory of RKHS enables us to represent a general nonlinear function as a linear weight vector **Ω** in the feature space.

#### 2.2.1 State Space Representation in the Reproducing Kernel Hilbert Space

From the perspective of dynamical systems, recurrent networks are essential for learning time structures and temporal dependencies in data. We are interested in estimating the hidden states recursively via the sequence of observations or measurements dependent on the state. The theory of RKHS allows us to construct a nonlinear state-space model with linear weights in the functional space.

First, we define a dynamical system in terms of a general continuous nonlinear state-transition and measurement functions, **f** (⋅, ⋅) and **h** (⋅), respectively,

$$\begin{array}{ll} x_{i} & =f\left(x_{i-1},u_{i}\right), \end{array}$$ (5)

$$\begin{array}{ll} y_{i} & =h\left(x_{i}\right), \end{array}$$ (6)

where

$$\begin{array}{ll} f\left(x_{i-1},u_{i}\right) & \overset{\Delta }{=}{\left[f^{\left(1\right)}\left(x_{i-1},u_{i}\right),\ldots ,f^{\left(n_{x}\right)}\left(x_{i-1},u_{i}\right)\right]}^{T}\\ & ={\left[x_{i}^{\left(1\right)},\ldots ,x_{i}^{\left(n_{x}\right)}\right]}^{T}, \end{array}$$ (7)

$$\begin{array}{ll} h\left(x_{i}\right) & \overset{\Delta }{=}{\left[h^{\left(1\right)}\left(x_{i}\right),\ldots ,h^{\left(n_{y}\right)}\left(x_{i}\right)\right]}^{T}\\ & ={\left[y_{i}^{\left(1\right)},\ldots ,y_{i}^{\left(n_{y}\right)}\right]}^{T}, \end{array}$$ (8)

with input vector $u_{i}\in R^{n_{u}}$, state vector $x_{i}\in R^{n_{x}}$, and output $y_{i}\in R^{n_{y}}$, where they have independent dimensionality or degrees of freedom, with the parenthesized superscript ^(*k*)^ denoting the *k*-th column of a matrix or the *k*-th component of a vector. To illustrate the input-agnostic property of a SPARK formulation, we first describe the KAARMA algorithm using a generic input sequence (real vector) **u**_*i*_, then change it for pulse trains in Section 2.3, which essentially amounts to a simple substitution of the reproducing kernel.

For simplicity, we express the dynamical system defined in Eqs 5, 6 using a new hidden state vector

$$\begin{array}{l} s_{i}\overset{\Delta }{=}\left[\begin{array}{c} x_{i}\\ y_{i} \end{array}\right]=\left[\begin{array}{c} f\left(x_{i-1},u_{i}\right)\\ h◦f\left(x_{i-1},u_{i}\right) \end{array}\right], \end{array}$$ (9)

$$\begin{array}{l} y_{i}=s_{i}^{\left(n_{s}-n_{y}+1:n_{s}\right)}=\underset{I}{\underset{︸}{\left[\begin{array}{cc} 0 & I_{n_{y}} \end{array}\right]}}\left[\begin{array}{c} x_{i}\\ y_{i} \end{array}\right], \end{array}$$ (10)

where ◦ is the function composition operator, **0** is an *n*_*y*_ × *n*_*x*_ zero matrix, and $I_{n_{y}}$ is the *n*_*y*_ × *n*_*y*_ identity matrix. By concatenating the original state vector **x**_*i*_ with the output **y**_*i*_, we create an augmented state vector $s_{i}\in R^{n_{s}}$, i.e., *n*_*s*_ = *n*_*x*_ + *n*_*y*_. Using this expression, the measurement equation simplifies to the selector matrix $I\overset{\Delta }{=}\left[\begin{array}{cc} 0 & I_{n_{y}} \end{array}\right]$.

Using the new state variable **s**, we define the following equivalent transition function **g** (**s**_*i*−1_, **u**_*i*_) = **f** (**x**_*i*−1_, **u**_*i*_), and Eqs 9, 10 becomes

$$\begin{array}{l} x_{i}=g\left(s_{i-1},u_{i}\right), \end{array}$$ (11)

$$\begin{array}{l} y_{i}=h◦g\left(s_{i-1},u_{i}\right). \end{array}$$ (12)

To model a dynamical system with general continuous nonlinear transition and measurement functions, **g** (⋅, ⋅) and **h**◦**g** (⋅, ⋅), respectively, we first map the input vector **u**_*i*_ and the augmented state vector **s**_*i*_ into two separate RKHSs as $\phi \left(u_{i}\right)\in H_{u}$ and $\varphi \left(s_{i}\right)\in H_{s}$, respectively. Next, the state-space model defined by Eqs 11, 12 can be rewritten as a set of weights (i.e., functions in the input space) in the joint RKHS $H_{su}\overset{\Delta }{=}H_{s}⊗H_{u}$, using the representer theorem, as

$$\Omega \overset{\Delta }{=}\Omega_{H_{su}}\overset{\Delta }{=}\left[\begin{array}{c} g\left(\cdot ,\cdot \right)\\ h◦g\left(\cdot ,\cdot \right) \end{array}\right],$$ (13)

where ⊗ denotes the tensor-product operator. The features in the joint tensor-product RKHS are defined as

$$\psi \left(s_{i-1},u_{i}\right)\overset{\Delta }{=}\varphi \left(s_{i-1}\right)⊗\phi \left(u_{i}\right)\in H_{su},$$ (14)

and the tensor-product kernel is defined as

$$\begin{array}{l} {\left\langle \psi \left(s,u\right),\psi \left(s^{\prime },u^{\prime }\right)\right\rangle }_{H_{su}}\overset{\Delta }{=}K_{a_{su}}\left(s,u,s^{\prime },u^{\prime }\right)\\ =\left(K_{a_{s}}⊗K_{a_{u}}\right)\left(s,u,s^{\prime },u^{\prime }\right)\\ =K_{a_{s}}\left(s,s^{\prime }\right)\cdot K_{a_{u}}\left(u,u^{\prime }\right). \end{array}$$ (15)

Here, data samples are evaluated using inner products or similarity measures. We can view the tensor-product kernel in Eq. 15 as an analogue to a soft-valued logical AND operator for joint similarity, e.g., to reach a desired next state requires both the proper input AND the right previous state.

Finally, combining Eqs 10–12, 13 the kernel state-space model (SSM) becomes

$$\begin{array}{l} s_{i}=\Omega^{T}\psi \left(s_{i-1},u_{i}\right), \end{array}$$ (16)

$$\begin{array}{l} y_{i}=Is_{i}. \end{array}$$ (17)

**FIGURE 2:**
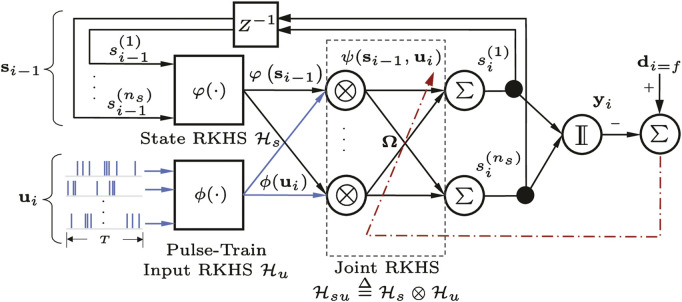
A kernel adaptive autoregressive-moving-average (KAARMA) network operating on multichannel pulse trains.

Figure 2 illustrates a simple kernel adaptive ARMA model operating on multichannel pulse trains. In general, the states variables **x**_*i*_ are hidden from the observer, and a desired output **d**_*i*_ may not be available at every time step, e.g., a deferred desired output value **d**_*f*_ or sequence label for the entire times series only appears at the final indexed step *i* = *f*.

In this paper, we are interested in learning and modeling the dynamics of an unknown system where the only information we know during training are the sequence labels, i.e., **y**_*f*_ = ±1 for a positive or negative example of a target class of sequences. This is an inference task, as opposed to a prediction problem. There is no predicting the next input in a sequence as is in the conventional frame-based approach of an HMM. The network either accepts or rejects the entire time series at the conclusion of each sequence. Compared to prediction problems, this is more difficult because we don’t have complete classification knowledge of every possible subsequence (i.e., when inference and prediction are equivalent). On the other hand, this formulation is much more useful and versatile, since a dynamical model makes no assumption on the sequence length *f*, i.e., it can operate on sequences of arbitrary duration. To tackle this problem, we will take a grammatical-inference approach. The state transition and measurement functions can be parametrized as the weights of a fully connected recurrent network and adapted by backpropagating the label error from the end of each input sequence.

Parameter adaptation for the linear state model is well understood, and the famed Kalman filter is an efficient recursive estimator that can update in real time. However, linear state model is not universal, i.e. solutions with small error are possible only when the desired response exists in the span of the input space (Haykin, 1998). Past research on dynamical modeling of complex nonlinear spatiotemporal signals such as speech demonstrates that the linear dynamical model is not competitive with the HMM statistical model. On the other hand, the theory of RKHS enables classical linear methods to produce general nonlinear solutions, and by operating in a potentially infinite-dimensional function space, we are freed from the limited expressiveness of the input space and model. The weights **Ω** in the RKHS can be learned using stochastic gradient descent, for a more detailed discussion on the adaptive update procedure with each incoming input sample, please refer to the full KAARMA derivation (Li and Príncipe, 2016). More recently, we have extended Bayesian filtering to the functional space in (Li and Principe, 2019).

The KAARMA algorithm achieves the best of both worlds: it preserves the simplicity of a linear dynamical model and features the universal property of functional spaces. This flexibility makes it ideally suited for applications involving multiple signal modalities and with different time scales, such as computational neuroscience applications using either local field potentials, spike trains, or a combination of both. The speech recognition application exemplifies a statistical learning approach to working with multichannel pulse trains, which improves the biorealism of the approach and allows us to take advantage of the energy-efficient and resource-conscious pulse information encoding and processing. Versatility is maximized by ensuring data-type interchangeability. The essential building block for designing the pulse-based KAARMA is therefore the kernel, which will be discussed next.

### 2.3 Reproducing Kernel Hilbert Space for Pulse Trains

A pulse train is represented as a sequence of *M* asynchronous, ordered spike timings, i.e., $P^{\left(i\right)}=\left\{t_{m}\in T:m=1,\ldots ,M\right\}$ in the interval T=[0,T], which can be interpreted as a realization of an underlying stochastic point process *i* with conditional intensity function $\lambda \left(t|H_{t}^{\left(i\right)}\right)$, time coordinate t∈T=[0,T], and the history of the process up to time *t* as $H_{t}^{\left(i\right)}$. The major challenge in pulse signal processing is that pulse trains are devoid of a natural algebra. To overcome this, we must first establish a space with the necessary properties for computation. Our approach is to define an appropriate reproducing kernel function on pulse trains that captures nonparametrically the instantaneous time structures and the variability of the event-based pulses. Once we define such a positive-definite or Mercer kernel for pulses, it maps the pulse trains into a Hilbert space of functions, allowing classic linear signal processing techniques to be directly applied via the *kernel trick*.

To define the joint tensor-product RKHS for pulse-based KAARMA implementation, we choose the Schoenberg kernel (Park et al., 2012), a binless, universal, nonlinear spike-train kernel. It uses the conditional intensity functions to compute similarity between two temporal point processes and is biologically-inspired. The Schoenberg kernel features three key advantages over other spike-train kernels: 1) defines an injective mapping 2) embeds neural responses of arbitrary stochasticity as the sample mean in the RKHS, and 3) approximates arbitrary spike-train function as a universal kernel (Park et al., 2013). For two conditional intensity functions $\lambda \left(t|H_{t}^{\left(i\right)}\right)$ and $\lambda \left(t|H_{t}^{\left(j\right)}\right)$, the Schoenberg kernel is defined as

$$K_{a_{\lambda }}\left(\lambda \left(t|H_{t}^{\left(i\right)}\right),\lambda \left(t|H_{t}^{\left(j\right)}\right)\right)\overset{\Delta }{=}\exp\left(-a_{\lambda }\int_{\tau }{\left(\lambda \left(t|H_{t}^{\left(i\right)}\right)-\lambda \left(t|H_{t}^{\left(j\right)}\right)\right)}^{2}dt\right),$$ (18)

with pulse-train kernel parameter *a*_*λ*_ > 0 (Paiva et al., 2009; Park et al., 2012; Dura-Bernal et al., 2016). For a given pulse train, we can estimate its conditional intensity function by convolving the pulse timings *t*_*m*_ with a smoothing function *g*(*t*) as

$$\hat{\lambda }\left(t\right)=\overset{M}{\underset{m=1}{\sum }}g\left(t-t_{m}\right),\left\{t_{m}\in T:m=1,\ldots ,M\right\}.$$ (19)

We can use this kernel to compute the inner product between a pair of pulse trains of the same duration *T* and arbitrary spike counts, either from a single integrate-and-fire converter taken at different times, or from two different IFCs in the same time window. For speech recognition, we are interested in quantifying the difference in the conditional intensity function estimates or temporal structures of the same pulse train or IFC channel across time. For multichannel pulse input, the similarity measure can be summed or averaged over all channels. Specifically, the multichannel pulse trains are segmented into smaller sub pulse trains of a fixed duration, similar to conventional speech segmentation using a fixed frame size and rate but without feature extraction. Figure 2 shows a KAARMA network operating directly on multichannel pulse-train frames of duration *T* at every discrete time step.

For simplicity but without the loss of generality, the rectangular smoothing function $g\left(t\right)=\frac{1}{T}\left(U\left(t\right)-U\left(t-T\right)\right)$ is used to estimate the conditional intensity function, with the Heaviside step function *U*(*t*) and $T\gg \bar{\Delta \tau }$, the average inter-pulse interval. Here, the pulse-train distance is defined on the precise pulse timings from each ordered set and agnostic to the pulse count. When the number of pulses are different, we pad the pulse train with the fewer count with the interval time or frame size *T*. One way to conceptualize this is that when two temporal point processes are similar, their pulse timings become closer or synchronized, reducing the pair-wise distance.

### 2.4 Kernel Adaptive AutoRegressive-Moving-Average Chain and Directional Learning

For learning long-term dependencies, a simple technique is to partition the input sequence into a fixed number of smaller sequences (length of each subsequence is not fixed) and train a chain of KAARMA networks in cascade, each responsible for a different ordered region of the input signal (Li and Príncipe, 2018b). We treat each ordered partition as a different grammar, and train a separate KAARMA network for each, but use the same class label. The overall recognition probability becomes the product of individual soft-max scores from each network.

Unlike static patterns which operate locally, reversing the sequence order generates a new dynamical system or complementary grammar that can be combined with the original to improve classification performance. Here, we combine the results of two KAARMA chains trained on the same sequences in the forward, left-to-right temporal direction (L2R or → with contextual information constructed from the past) and the backward, right-to-left ordering (R2L or ← with future context), by simply multiplying their softmax scores to derive a bi-directional (*⇌*) system with enhanced recognition rate.

### 2.5 Rule Extraction for Sparse-Embodiment Deployment in Edge Computing

In formal language theory (Harrison, 1978), deterministic finite automata recognize regular grammars in the Chomsky hierarchy (Chomsky, 1956) and can operate in either language generation or validation mode. A DFA is formally defined as a 5-tuple: $A=\left\langle Q,\Sigma ,\delta ,q_{0},F\right\rangle$, with a finite set of states **Q**, a finite input alphabet **Σ**, a state transition function *δ* where (*δ*: **Q** ×**Σ** → **Q**) maps the current state and input symbol to the next state, the initial state $q_{0}\in Q$, and a set of accepting (final) states *F* ⊆**Q**. A DFA can be implemented efficiently as a lookup table. For an input sequence or word *w* over the alphabet **Σ**, the DFA A recognizes *w* if it arrives at an accepting state after the last symbol, otherwise, *w* is deemed ungrammatical and rejected. The language L(A) is the set of all acceptable strings by A.

A grammar, on the other hand, is a 4-tuple: G=⟨N,T,P,S⟩, with disjoint finite sets of nonterminal symbols **N**, terminal symbols **T**, a set of production rules **P**, and the start symbol *S* ∈**N**. A grammar is *regular* if and only if every production rule in **P** is one of the following three forms: 1) B→a, 2) B→aC, or 3) *B* → *ϵ*, where *B* and *C* are in the nonterminal set **N** with *B* = *C* allowed, a∈T, and *ϵ* is the empty string. From A, one can easily construct a regular grammar such that L(G)=L(A). The grammar is the generative descriptor of a language while the corresponding DFA is the analytical descriptor.

A DFA or FSM models a discrete-time dynamical system (DTDS) in a discrete state space, where from an initial state, input sequence uniquely determine all the state transitions. From this perspective, DTDS identification can be viewed as a grammatical inference task: from a training set of positive and negative examples of sequences, infer the grammar satisfying all available samples. However, grammar induction is NP-complete (Gold, 1978). Heuristic algorithms were used in early research on DTDS, but were shown to scale poorly with the automaton size (Angluin and Smith, 1983). Since the 1940s, the relationship between FSM and recurrent neural networks (RNNs) has been studied extensively (McCulloch and Pitts, 1943). Minsky showed that RNNs can simulate any FSM (Minsky, 1967), followed by Siegelmann, for arbitrary Turing machine in real-time (Siegelmann and Sontag, 1995). In (Li and Príncipe, 2016), we showed that KAARMA networks can infer grammars efficiently using far fewer training samples than RNNs. Using a dynamical system approach with grammatical inference formulation, KAARMA captures the dynamics of the input sequences with a small set of attractors, which greatly facilitates the extraction of a finite state machine or lookup table from the trained network.

For real-valued data (infinite alphabet size), our objective is to generalize the input patterns and states to a set of prototypes, then use the trained dynamical system to enumerate and trace all possible next states and form a memory-based lookup table, which can be embedded in hardware and operated on the fly without performing complex computations. To derive a finite state representation from a trained KAARMA network, we first discretize the input and state spaces. The *curse of dimensionality* associated with high-dimensional state and input spaces can be easily avoided for kernel methods by clustering only parts of the space where data is available, i.e., subspace spanned by the training data. Using the spatial clustering method outlined in (Li and Príncipe, 2016, Algorithm 2), we introduce two quantization factors or distance-based clustering thresholds, *q*_input_ and *q*_state_, for the input space and the state space, respectively. The initial alphabets consist of the initial state and the first input vector. To form their respective alphabets **Q** and **Σ** directly from the training data, states (where next states are generated using the trained KAARMA network) and input points are introduced to the spatial clustering algorithm or quantizer one at a time. The Euclidean distances are computed with respect to all previous data centers or letters in the alphabets. If the minimum distance for the new data point is smaller than the quantization factor, the dictionary or alphabet is kept the same; otherwise, it is added to the corresponding alphabet. This sparsification technique has been previously used to train KAARMA networks using significantly fewer data points than available (Li and Príncipe, 2016).

After we have fixed the input and state alphabets of the FSM by running the entire training data through the trained KAARMA network and applying spatial clustering to all incoming data points using thresholds *q*_input_ and *q*_state_, we can map out the state transition table (*δ*: **Q** ×**Σ** → **Q**) for any input-state pairs. Again, this is accomplished by processing all input-state pairs in the alphabets **Σ** and **Q** through the trained KAARMA network and indexing the nearest Euclidean neighbor in the state alphabet **Q** for the next state output. Once a state-transition table is extracted, we can further minimize the size of the state alphabet by removing unreachable states and merging non-distinguishable states to form the minimal automaton, as shown in (Li and Príncipe, 2016).

## 3 Simulation Results

Speech is a perfect application to demonstrate the competitive advantage of SPARK to quantify time structure instead of conventional Markov Chain statistical approach, but our proposed SENSA framework applies to all locally stationary signals. Statistics have difficulty coping with temporal information because a random process is a family of random variables over time that violate the independent and identically distributed (i.i.d.) property, the simplifying assumption that enables statistics for real-world phenomena, e.g., Bayesian filter requires time-consuming conditional expectations for quantifying the corresponding time structure.

### 3.1 Automatic Speech Recognition System Using Finite State Machines

To demonstrate our proof-of-concept implementation, we used the TI-46 corpus of isolated digits for benchmarking the KAARMA-based SPARK classifiers. This dataset consists of spoken words from eight male and eight female English speakers, each uttering the digits, “zero” through “nine”, 26 times, at sampling frequency of 16 kHz. Out of the 4160 total recordings, 25 out of the 26 utterances in each digit-speaker pair (or 4000 samples) were used in the subsequent multispeaker experiments. These utterances were further randomly partitioned into a training set of 2,700 utterances with an equal number of male/female utterances and digits (i.e., 270 total utterances per digit with half from female speakers) and a testing set of 1,300 utterances with an equal number of male/female utterances and digits.

In conventional automatic speech recognition (ASR) front-end using real-valued Mel-frequency cepstral coefficients (MFCCs) (Davis and Mermelstein, 1980), the following preprocessing and feature engineering were performed: Hamming window each input frame and apply a first-order pre-emphasis filter (*α* = 0.95), next compute the magnitude spectrum using the discrete Fourier transform (DFT) then scale by a Mel-filterbank, lastly obtain the MFCCs from the log-compressed and discrete cosine transformed energy output. A total of 13 MFCCs were computed, but only the last 12 coefficients were used to represent the static speech features in each frame of 25 ms at a rate of 100 frames-per-second (fps), i.e., at 10 m s intervals or 60% overlap.

For a human-engineered solution, we can expect good performance using only a 12-dimensional feature vector. Here, we demonstrate the feasibility of performing the same ASR task using pulse trains where information is encoded with high efficiency in the precise pulse timings (events over time) without further feature engineering, instead of working with waveforms where information is encoded in the amplitude. Increasing the number of pulse channels should improve the recognition accuracy, but our goal is to establish a baseline performance using only 12 channels of pulse-train input (same number of effective channels as the Mel-filterbank setup in conventional ASR). To generate the biologically-inspired pulse trains, we applied a 12-filter gammatone filterbank (Patterson et al., 1987) with equally spaced center frequencies (50 Hz–8 kHz) on the equivalent rectangular bandwidth (ERB)-rate scale to each speech signal, which mimics the cochlea in the human auditory system. To convert the 12-channel filtered output into a sparse representation or pulse trains, we normalized the maximum absolute amplitude to 4 *μA* and used integrate-and-fire neurons or IFCs (one per channel) with refractory current and spike-rate adaptation (SRA) (Gerstner and Kistler, 2002). Table 1 summarizes the parameters used to convert speech signal into pulse trains.

**TABLE 1:** Integrate-and-fire converter (IFC) parameters.

Parameter Description	Value
Membrane resistance, *R*_*m*_	10 MΩ
Time constant, *τ*_*m*_	10 ms
Spike threshold, *Vth*	−55 mV
Spike delta, *V*_*spike*_	500 mV
Reversal potential for SRA, *E*_*K*_	−200 mV
Reset potential, *V*_*reset*_	−80 mV
SRA time constant, *τ*_*sra*_	200 ms
Increase in SRA per spike, Δ_*sra*_	5 nS
Time for refractory conductance to decay, *τ*_*ref*_	2 ms
Increase in refractory conductance per spike, Δ_*ref*_	200 nS

**FIGURE 3:**
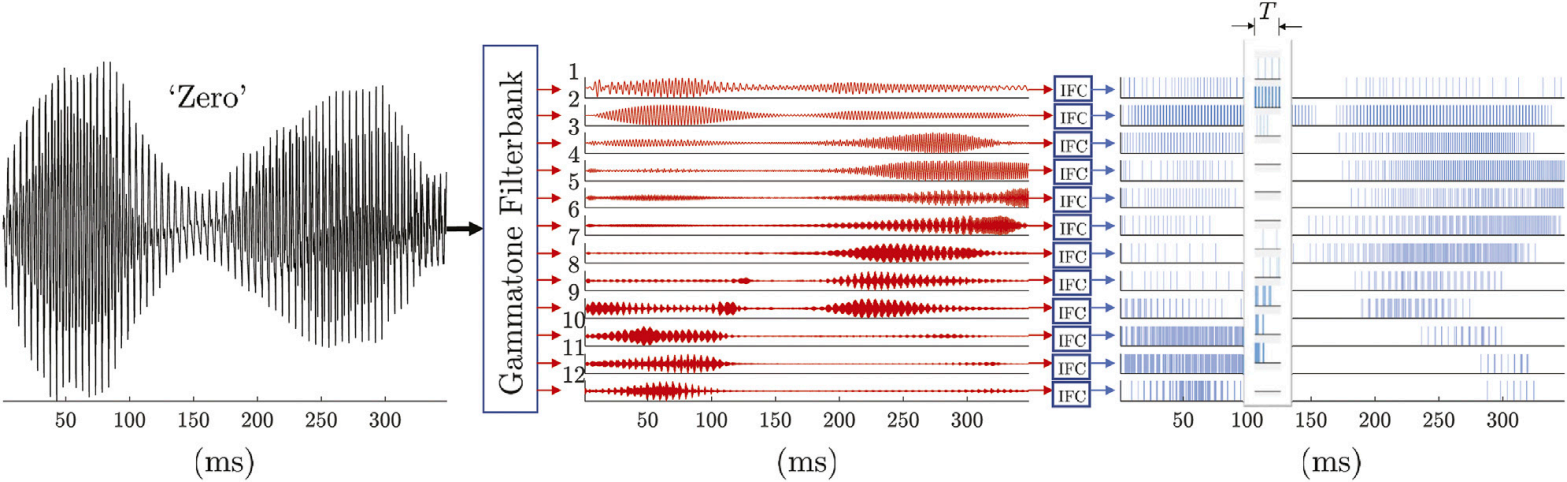
Pulse train front-end for speech recognition: speech signal (sampled at 16 kHz) first passes through a 12-channel gammatone filterbank with center frequencies equally spaced between 50 Hz and 8 kHz on the ERB-rate scale, then converted into pulse trains using leaky integrate-and-fire neurons or IFCs. This encodes the time series into a biologically-inspired, highly-efficient sparse representation with mean spike count per frame (*T* = 25 ms) ranging from 0.42—25.49 across different channels and digits.

For training, pulse-train sequences were labeled ±1 based on the target digit class and segmented into frames the same way as the MFCCs using 25-ms frames at 100 fps. Unlike the MFCCs, we directly fed the multichannel pulse trains in each time frame as features to the pulse-based KAARMA networks without further feature engineering (i.e., temporal coding) with model parameter values listed in column 2 of Table 2; Figure 3 shows the pulse-based front-end for speech processing.

**TABLE 2:** KAARMA parameters.

Parameter Description	Value	
	(Temporal Coding)	(Rate Coding)
Spike-train kernel parameter, *a*_*λ*_	1	−
RBF kernel parameter, *a*_*u*_	−	5
Hidden-state kernel parameter, *a*_*s*_	4	5
Learning rate, *η*	0.1	0.1
Kernel-distance quantization threshold, *q*	0.25	0.55

To reduce the bias from data imbalance using the one-vs-all approach for classification in each word model, the positive class (10% of the data for each target digit) was replicated 3 times in the training set with random placement. The final ASR system consists of 10 word models, one for each digit. During processing, the word model with the highest confidence (largest positive final output value) is selected as the predicted class. To improve the recognition performance and alleviate the need to learn long term dependencies, each utterance is partitioned into five equal segments, this is similar to the 5-state HMM set up. Depending on the individual utterance, each segment can contain one or many speech features, with the total number of segments fixed at 5. In this topology, each word model consists of five smaller KAARMA networks operating in cascade. One 5-network KAARMA chain was used to model each of the ten digits and trained for a single epoch only. The model parameters were not fully optimized over their respective ranges to reduce overfitting. Table 3 summarizes the results. Since HMM has no native support for pulse input, the pulse count in each frame (i.e., rate coding) was used to compute the firing rate and formed a continuous-valued 12D feature vector across all channels. KAARMA networks, on the other hand, are agnostic to input representation and support both data types, with rate-coding parameter values shown in column 3 of Table 2.

**TABLE 3:** Comparisons of KAARMA chain classifiers with HMMs using an equivalent number of states and a mixture of eight Gaussians per state. Only 12 MFCC coefficients were used, without log energy and time derivatives. Similarly, only 12 channels of pulse trains were used.

5-State HMM				
Input Type			Training (%)	Testing (%)
MFCC			98.74	98.00
Pulse Train	Rate Code		93.74	93.23

**5-Network KAARMA Chain**				
Input Type			Training (%)	Testing (%)
MFCC	Sequence Ordering	L2R →	99.33	98.62
		R2L ←	*99.48*	*98.31*
		*⇌*	***99.78***	***99.08***
Pulse Train	Rate Code	L2R →	99.04	91.85
		*⇌*	**99.56**	94.54
	Temporal Code	L2R →	96.70	93.54
		R2L ←	97.33	92.46
	(Schoenberg Kernel)	*⇌*	98.56	**95.23**

**FIGURE 4:**
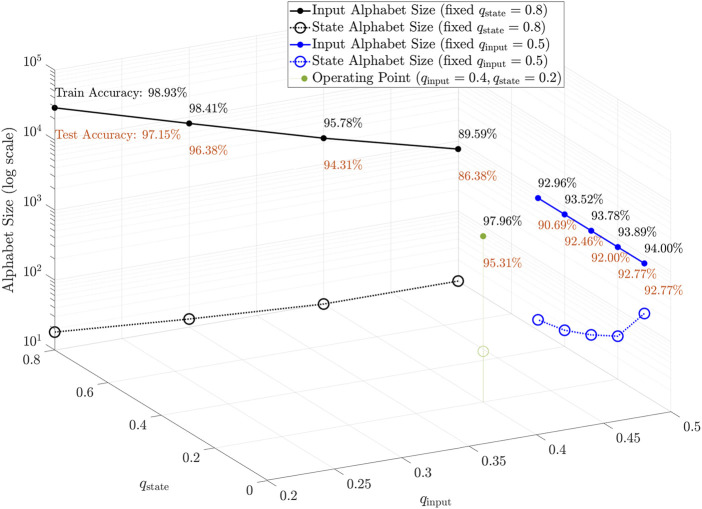
Performance as a function of quantization thresholds. Input alphabet is uniquely determined by its quantization factor *q*_input_ (e.g., the solid blue line shows a constant alphabet size of 770 for a fixed threshold *q*_input_ = 0.5, regardless of state quantization values), whereas the state alphabet is a function of both the input and state quantizations (e.g., dashed black line shows a reduced state alphabet as the input quantization increases, even with the state quantization fixed at *q*_state_ = 0.8). Certain states become unreachable when the data resolution is reduced. Clearly, latent states are parsimonious descriptors of nonlinear dynamics: good recognition accuracies are maintained using only a small subset of the original state space points, i.e., most state variables orbit a few attractors.

Table 4 shows the training and testing set performances of finite state behavior using different clustering thresholds. The input and state dictionary sizes are determined from the training set by the respective quantization factors, and fixed for the testing set. As we increase the *q* thresholds, sparsity is also increased, as more and more data points are clustered into the same prototype. Figure 4 shows the performance as a function of quantization thresholds. Recognition accuracies are inversely proportional to the *q* values, as with increased sparsity, data resolution is decreased. Nonetheless, we can still retain high recognition accuracies using only a very small subset of the original state space points. We see that for a trained KAARMA chain, the extracted finite state behavior is largely determined by the input alphabet size. Reducing the input resolution limits parts of the state space that is utilized.

**TABLE 4:** Performance of discretized 5-network KAARMA chains using MFCCs for the forward, left-to-right (L2R or →) sequence processing.

Threshold		Input Alphabet	State Alphabet	Training	Testing
*q*_input_	*q*_state_	**Size**	**Size**	**Accuracy** (%)	**Accuracy** (%)
0.2	0.8	28,903	18	98.93	97.15
0.3	0.8	8,116	13	98.41	96.38
0.4	0.8	2,344	10	95.78	94.31
0.5	0.8	770	10	89.59	86.38
0.5	0.5	770	14	92.96	90.69
0.5	0.4	770	17	93.52	92.46
0.5	0.3	770	25	93.78	92.00
0.5	0.2	770	41	93.89	92.77
0.5	0.1	770	148	94.00	92.77
0.4	0.2	2,344	53	97.96	95.31

**FIGURE 5:**
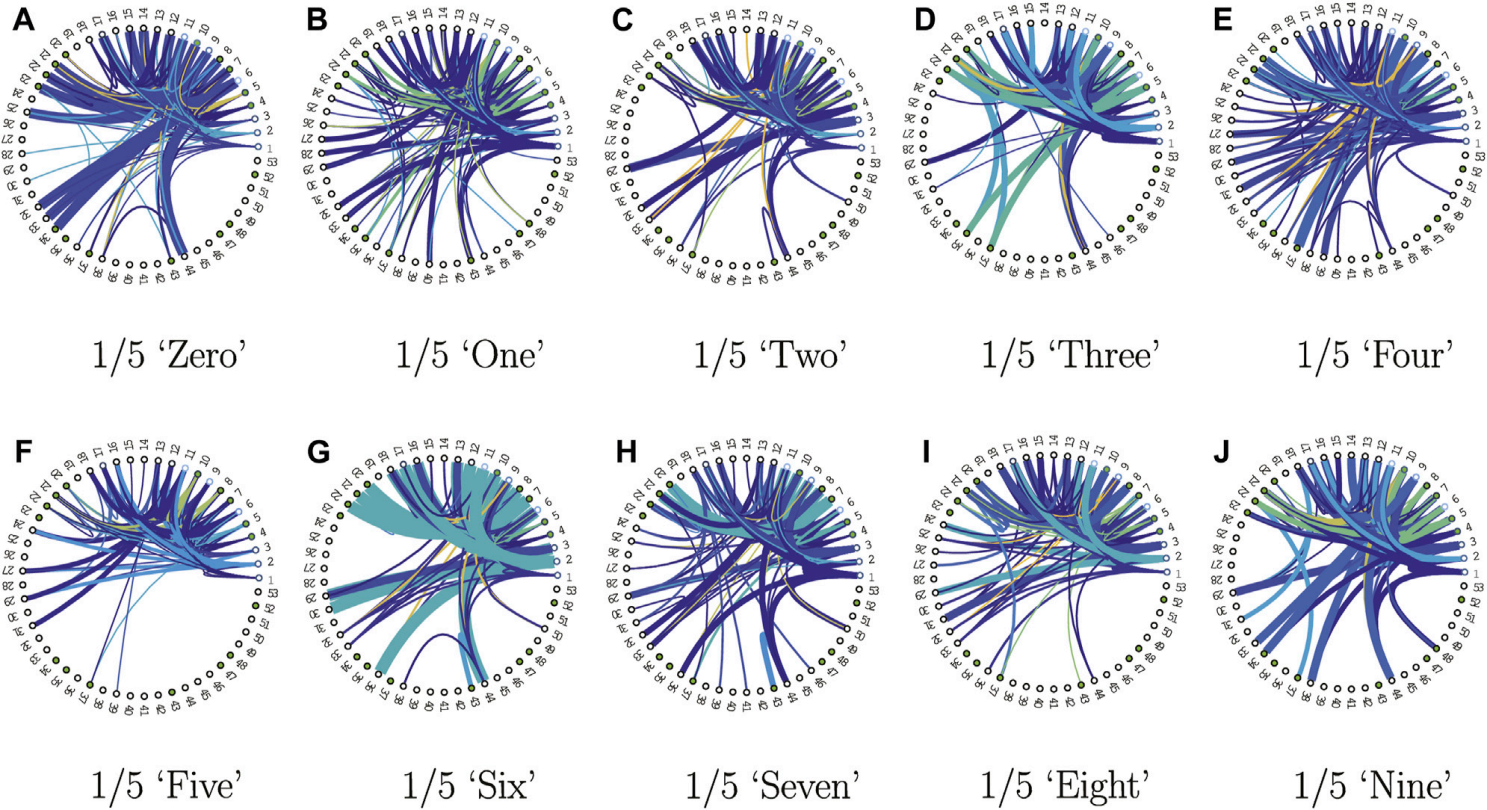
State connectivity graphs for the first networks of the ten SPARK word models, with training accuracy of 97.96% and testing accuracy of 95.31%: **(A)** corresponds to the first network of the 5-network KAARMA chain (denoted by 1/5) word model for the digit “zero”, etc. Accept states are marked by filled green dots. Only the upper-triangular destination-state connections are considered in the connectivity graphs, with self-transitions ignored.

**FIGURE 6:**
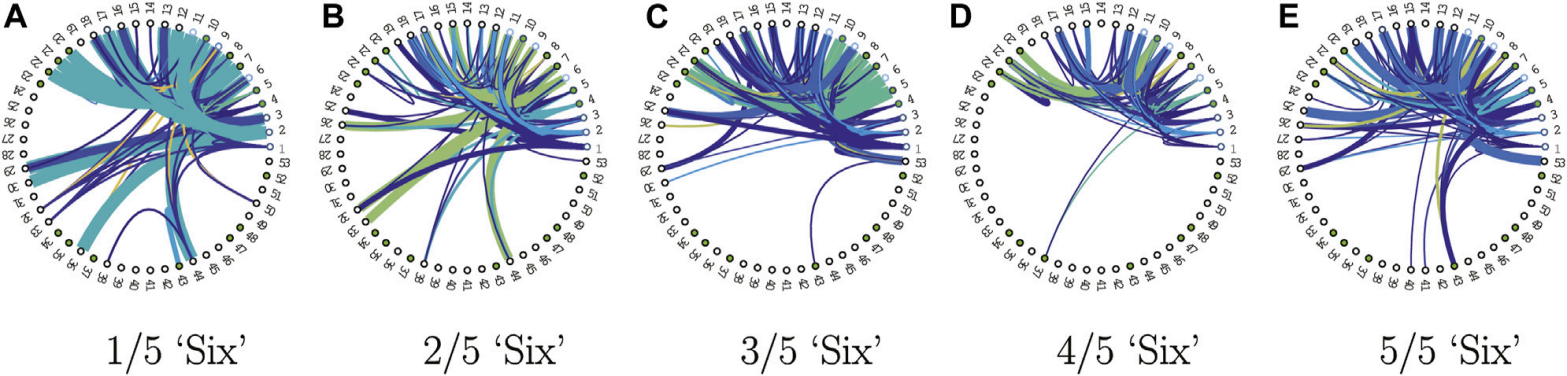
State connectivity graphs for the 5-network KAARMA chain corresponding to the word model “six”.

Without loss of generality, a reasonable tradeoff between performance and sparsity is achieved using *q*_input_ = 0.4 and *q*_state_ = 0.2, as shown in Table 4, resulting in a fixed input alphabet size of 2,344 and a fixed state size of 53, with a good test set accuracy of 95.31*%*. We visualized the finite state behavior or lookup tables of the quantized networks in Figures 5, 6. Since the input alphabet size 2,344) is relatively large compared to the number of states 53) used, we used the following compact state transition or connectivity graphs, with only undirected transitions between pairs of states shown. Each of the 10 word models, corresponding to the digits “zero” through “nine”, consists of five FSMs or lookup tables, each responsible for an ordered partition of arbitrary length. States are expressed sequentially in a radial graph, with the first state or **s**_1_ at the 0-radian location. Accept states are marked by filled green dots. Since each lookup table maps out the directional next-state transition for all possible state-input pairs, without loss of generality and for clarity, only the upper-triangular destination-state connections are considered in the connectivity graphs, with self-transitions ignored. The width of the edge is proportional to the strength or frequency of state transition, and the colormap indicates the input composition. Figure 5 shows all the first network (out of five in the KAARMA chain) FSMs for each of the 10 word models, while Figure 6 shows all five networks for the word model “six”. These connectivity graphs clearly illustrate that while all the FSMs share the same state and input alphabets, they exhibit distinctly different dynamics. For example, not all states serve as destination or next state.

Similarly, we can generate lookup tables for pulse trains, as shown in Table 5. Overall, there is a decrease in performance when using spike-based front-ends, as shown in Table 3. This is not only a testament to the ingenuity and prevalence of the human-engineered MFCC as the *de facto* speech feature, but also to the fact that the focus of this research is not to optimize the feature engineering, but rather to demonstrate, as a proof-of-principle, that a simple lookup table achieves competitive result using pulse trains. Despite this drop in performance, we have shown that pulse-train inputs are more robust to noise than MFCCs (Li and Príncipe, 2018b). Like the case involving MFCCs, good performances are obtained using FSMs operating on pulse trains, by tuning the clustering threshold values. We observe that reversing the pulse train sequence produced not only different grammars or automata, but also alphabets of different sizes using the same cluster thresholds. This is because the estimate for the conditional intensity function of a point process is now computed backwards, and for two pulse trains with different spike count, the padding is now on the opposite end, creating different, shifted pulse timing pairs. We see that a good performance of 94.54% testing accuracy (−0.69% from the unquantized pulse-train performance) is achieved using fewer than 7,000 input patterns and a state size of fewer than 200.

**TABLE 5:** Performance comparison of SPARK (5-network KARMA chains) using pulse trains with other spike-based methods.

SPARK (TI-46, 16 speakers)											
Threshold		Input Alphabet Size		State Alphabet Size		Training Accuracy (%)			Testing Accuracy (%)		
*q*_input_	*q*_state_	→	←	→	←	→	←	*⇌*	→	←	*⇌*
0.30	0.4	1,353	1,362	34	45	80.85	86.96	90.59	79.54	85.38	90.54
0.25	0.4	2,968	2,960	34	45	87.07	91.56	94.37	83.77	89.23	92.92
0.20	0.4	6,951	6,905	34	45	91.15	93.00	95.41	87.85	91.54	93.69
0.20	0.2	6,951	6,905	150	191	94.37	94.26	**96.37**	90.62	92.38	**94.54**

**Pulse or Spike Front-End for Spoken Digit Recognition (TI-46)**											
Model (→)				Input Spike Channels	Network Size				Speakers		Accuracy (%)
5-Network KAARMA Chain				12	1880_center_ × 5_network_ × 10_word_				16		93.54
SENSA-SPARK				12	1_table_ _lookup_ × 5_network_ × 10_word_				16		90.62
Digital LSM Zhang et al. (2015)				83	83_neuron_ × 135_neuron_ × 10_neuron_				16		92.30
SWAT SNN Wade et al. (2010)				180	180_neuron_ × 5040_neuron_ × 10_neuron_				8		95.25
LSM Verstraeten et al. (2005)				39	39_neuron_ × 1232_neuron_ × 10_neuron_				5		95.50

The aim of SENSA is to trade a small performance drop in the digitization of the hardware-deployed model for orders of magnitude decrease in power consumption and footprint. Compared to the SNN methods with adaptive weights, the only computation involved to operate these automata over pulse trains is indexing: locate the closest pulse pattern in the input alphabet, which can be optimized in hardware since the similarity metric is event driven and accumulated over time with each pulse timing, then retrieve the next state automatically from memory.

### 3.2 Field Programmable Gate Array Finite State Machines Implementation

Once the finite state machines are extracted, the hardware implementation is very straightforward. The design of the speech recognition automata was done in Verilog, with the functional correctness of the design verified by comparing its results with that obtained from the software implementation. Without loss of generality, we describe the design implement for the first network of the word model “zero” in the 5-network KAARMA chain for MFCC input. Since the pulse train implementation is also memory based, we will derive a general formula for the power consumption and footprint based on the lookup table size.

First, indexing of the data is performed using the Euclidean distance between the input vector and the input alphabet, which determines the location of the closest input symbol in the lookup table or FSM. With this index and the current state (initial state is fixed for all lookup tables), we retrieve the next state from memory. The above set of steps are repeated until we reach the end of the input sequence. The final state determines the classification or recognition of the entire sequence based on its sign (accept/reject), since the output component of the state vector is trained using ±1 label.

**FIGURE 7:**
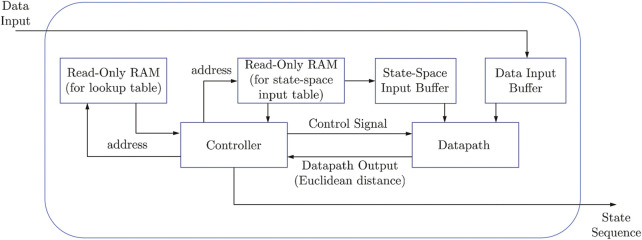
Top level schematic for FSM implementation in FPGA hardware using digital data.

Figure 7 shows the top-level schematic of the implementation in FPGA. The data for the lookup table (state transitions) with size 2,344 × 53 (input alphabet size by state alphabet size) and the input alphabet with size 12 × 2,344 (feature vector dimension by input alphabet size) are stored in read-only RAM. The lookup table contains the indices (1–53) of the next states, addressed by the input-state coordinates. In order to store these values in RAM, they are converted to binary representation using regular binary to decimal converter. The input array consists of 12D column vectors corresponding to each prototype in the input alphabet. To properly represent the values inside this array in binary, with the minimal number of bits, we first quantized them, then converted to binary. Here, the quantized values are represented with 8 bits, in the form of two’s complement to represent the positive and negative values. Binary representation of the input data is obtained in the same way as the input alphabet, using 8-bit binary.

**FIGURE 8:**
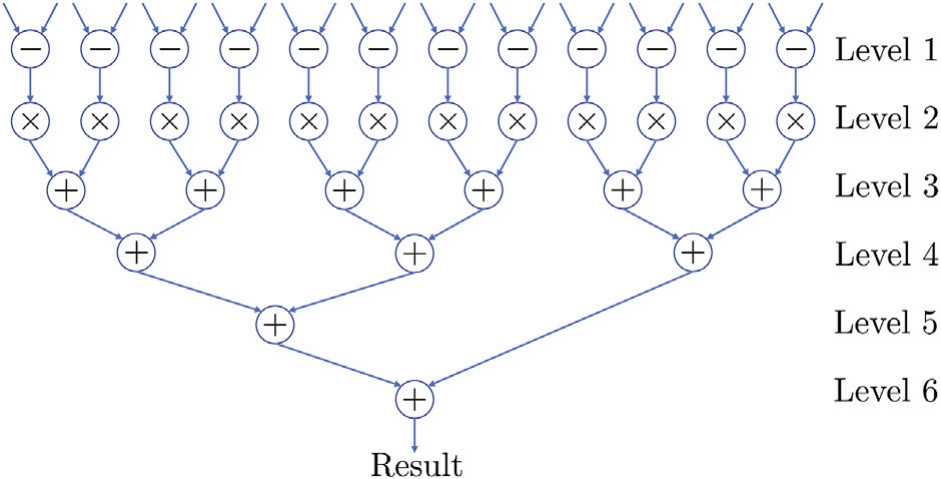
Multilevel datapath for computing the Euclidean distance between the input data (12D) and the input alphabet, which takes six clock cycles.

The only computationally expensive operation is locating the nearest neighbor of the current input in the alphabet using distance. We parallelized this operation by implementing a multileveled datapath, shown in Figure 8. Both inputs to the datapath are 12D vectors. Level 1 of datapath subtracts pairwise elements of both inputs simultaneously across all 12 dimensions. Level 2 squares the outputs obtained from the previous level. Levels 3, 4, and 5 aggregate the squares obtained from Level 2. Register level is present between the output of each level and the input of next level. Using this datapath structure, it takes six clock cycles to calculate the Euclidean distance between one pair of inputs. Note, the datapath can be pipelined to improve performance by increasing throughput.

The controller is implemented as a five-stage FSM, which generates control signals for datapath and read addresses for the RAMs. It outputs the sequence of state transitions for the given set of data inputs. The segregation of the computation and control function of the system makes it easier to accommodate future improvements such as pipelining the datapath, etc.

**FIGURE 9:**
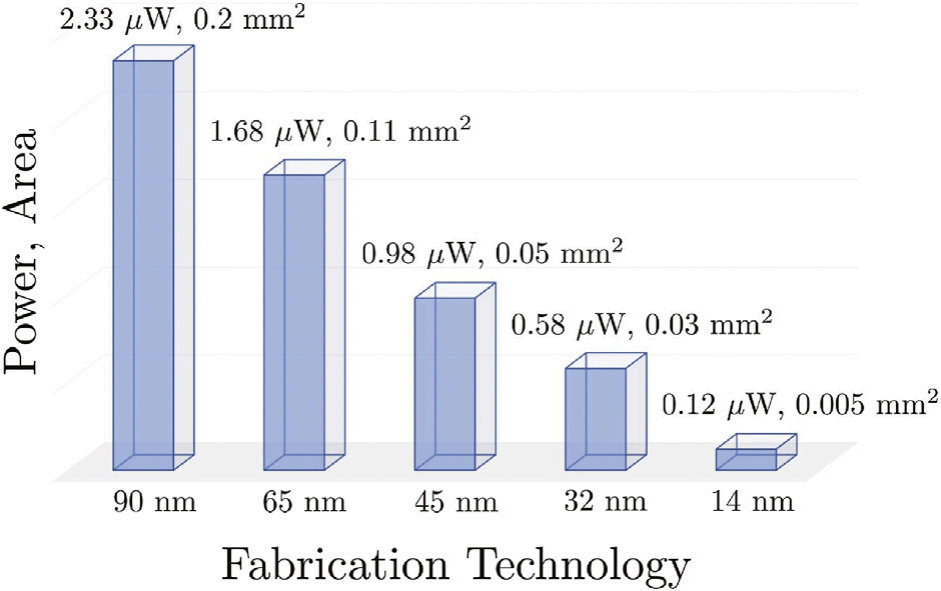
Power consumption and footprint scaling of a single FSM to more advanced fabrication technology.

For a single keyword acoustic model in FSM using conventional MFCC features, we obtained the following power and footprint measurements, shown in Table 6, using 10 kHz clock and SMIC 0.18 *μ*m process technology. When scaled to state-of-the-art fabrication technologies, we can expect sub *μ*W power consumption, effectively moving into the nW range, as shown in Figure 9. This is superior to industry standard for hardware-based keyword spotting using similar input. The power *P* and area *A* scaling are as follows

$$\begin{array}{ll} A_{L2} & =A_{L1}{\left(\frac{L2}{L1}\right)}^{2}, \end{array}$$ (20)

$$\begin{array}{ll} P_{L2} & =P_{L1}\left(\frac{L2}{L1}\right){\left(\frac{V_{L2}}{V_{L1}}\right)}^{2}, \end{array}$$ (21)

where *L*1 and *L*2 are the characteristic lengths of the two different processes, with *V*_180nm_ = 1.8 V, *V*_90nm_ = 1.2 V, *V*_65nm_ = 1.2 V, *V*_45nm_ = 1.1 V, *V*_32nm_ = 1 V, and *V*_14nm_ = 0.7 V. According to recently published research (28 nm CMOS with power consumption of 141 *μ*W), our figures are at least two orders of magnitude better than DL-based solutions (Zheng et al., 2019).

**TABLE 6:** Summary of power consumption and footprint of a single FSM or lookup table.

Lookup RAM Size (bits)	Power (*μ*W)	Area (mm^2^)
3,392*8	15.3	1.0
2,544*8	14.1	0.92
1,696*8	10.5	0.83

The hardware implementation for pulse trains is similar to that of real-valued feature vectors. Instead of using the Euclidean distance between two fixed-dimension vectors to locate the closest pattern in the input alphabet of an FSM, the distance between the estimated conditional intensity functions of the Schoenberg kernel is used. As discussed above, this amounts to the squared pairwise pulse-timing differences between two pulse trains, where the pulse train with the fewer pulse count is padded with the necessary number of pulse timings corresponding to the window duration. This can be computed piecewise in time, whenever a pulse is received.

To further reduce the power consumption and footprint, the hardware embedment can be implemented in an application specific integrated circuit (ASIC). In general, the front end of SENSA will consist of a filterbank with IFC at each output, converting the analog signal into sparse pulse representation, where the amplitude information is encoded in the inter-pulse interval. SPARK is then used to derive nonparametric solution directly from the multichannel pulse trains and embed the intelligence at the edge using automata. Another way to minimize each automaton is to limit the pulse patterns or prototypes in the alphabet by reducing the window or frame size. At sufficiently small frames, we will be operating at the resolution of a single pulse, i.e., binary alphabet with a value of either one (pulse) or zero (no pulse). We also envision a network of simple distributed SPARK embedded hardware performing in concert to solve more complex problems and make better intelligent decisions at the edge.

## 4 Conclusion

The SENSA framework leverages data-driven intelligence with biologically-inspired efficiency for downscaling complex machine learning solutions to deliver edge intelligence. As a proof-of-concept, we demonstrated the feasibility of SPARK (one specific formulation of SENSA using the theory of RKHS) to operate an automatic speech recognition system using only lookup tables. We can apply this methodology to other time series, not just limited to acoustic signal, using the appropriate analog-to-pulse converter. This creates countless opportunities for novel applications that benefit from ultra-low power, ultra-fast computation, and with improved noise robustness, particularly in delivering resource-constrained intelligence to the edge of IoT.

In the future, we will design and fully implement an ASR for isolated and continuous speech in hardware operating at the pulsing resolution of zeros and ones. Furthermore, we will implement and test reconfigurable FSMs in hardware to enable user customization on pre-trained models using one-shot or few-shot learning.

## Data Availability

Publicly available datasets were analyzed in this study. This data can be found here: https://catalog.ldc.upenn.edu/LDC93S9.
